# CD47: The Next Frontier in Immune Checkpoint Blockade for Non-Small Cell Lung Cancer

**DOI:** 10.3390/cancers15215229

**Published:** 2023-10-31

**Authors:** Asa P. Y. Lau, Sharon S. Khavkine Binstock, Kelsie L. Thu

**Affiliations:** 1 Laboratory Medicine and Pathobiology, University of Toronto, Toronto, ON M5S 1A8, Canada; 2 Keenan Research Centre for Biomedical Science, St. Michael’s Hospital, Toronto, ON M5B 1T8, Canada

**Keywords:** CD47, immune checkpoint inhibitors, non-small cell lung cancer, immunotherapy

## Abstract

**Simple Summary:**

Lung cancer is the leading cause of cancer-related death worldwide. Despite treatment advances, high rates of tumor recurrence emphasize the need for new therapeutic strategies. Tumors often acquire mechanisms to avoid detection by the immune system, allowing them to develop and metastasize. Immunotherapy is a type of treatment designed to overcome these mechanisms by reactivating the immune system to eliminate tumors. CD47 is a cell surface protein and marker of “self” expressed on cells throughout the body and prevents them from being “eaten” by cells of the immune system. Lung cancers exploit this “don’t eat me” signal by upregulating CD47 to evade the immune system, making it a promising therapeutic target. This review summarizes the roles of CD47 in tumor biology, its therapeutic potential in non-small cell lung cancer, and challenges that must be overcome to facilitate the clinical translation of CD47-targeted immunotherapy to improve lung cancer survival rates.

**Abstract:**

The success of PD-1/PD-L1-targeted therapy in lung cancer has resulted in great enthusiasm for additional immunotherapies in development to elicit similar survival benefits, particularly in patients who do not respond to or are ineligible for PD-1 blockade. CD47 is an immunosuppressive molecule that binds SIRPα on antigen-presenting cells to regulate an innate immune checkpoint that blocks phagocytosis and subsequent activation of adaptive tumor immunity. In lung cancer, CD47 expression is associated with poor survival and tumors with EGFR mutations, which do not typically respond to PD-1 blockade. Given its prognostic relevance, its role in facilitating immune escape, and the number of agents currently in clinical development, CD47 blockade represents a promising next-generation immunotherapy for lung cancer. In this review, we briefly summarize how tumors disrupt the cancer immunity cycle to facilitate immune evasion and their exploitation of immune checkpoints like the CD47–SIRPα axis. We also discuss approved immune checkpoint inhibitors and strategies for targeting CD47 that are currently being investigated. Finally, we review the literature supporting CD47 as a promising immunotherapeutic target in lung cancer and offer our perspective on key obstacles that must be overcome to establish CD47 blockade as the next standard of care for lung cancer therapy.

## 1. Lung Cancer and the Evolving Treatment Landscape

Lung cancer is the leading cause of cancer-related death worldwide and was estimated to cause over 600,000 deaths in 2022 in the United States alone [1,2,3]. Although the implementation of screening programs is expected to facilitate earlier detection and improved outcomes [2], nearly 50% of lung cancers are diagnosed at advanced and inoperable stages, which is a major contributing factor to the dismal 5-year net survival rate of ~20–22% [2]. Lung tumors are a heterogeneous group of malignancies that are broadly classified into small-cell lung cancer (SCLC) or non-small cell lung cancer (NSCLC), accounting for approximately 15% and 85% of all cases, respectively [4]. NSCLC is further classified into histological subtypes including adenocarcinoma (LUAD), squamous cell carcinoma, and large cell carcinoma, of which LUAD is the most common [5]. Within these subtypes, lung cancers exhibit extensive heterogeneity at the molecular level, and typically exhibit hundreds to thousands of DNA alterations due to nucleotide and chromosomal instabilities inherent to all subtypes [6,7,8]. These genomic instabilities fuel tumor evolution, drug resistance, and disease progression [9]. As a consequence, few patients experience durable responses to standard of care treatments, and therapeutic development continues to be a priority for improving lung cancer survival rates [10,11,12,13].

Traditionally, lung cancer has been treated with surgery, chemotherapy, radiation, and targeted therapy guided by disease stage, histological subtype, and genetic testing [14,15,16]. In 2015 and 2016, the treatment landscape for NSCLC was transformed by the approval of immune checkpoint inhibitors (ICIs) targeting PD-1 and PD-L1, immunosuppressive proteins that tumors exploit to evade the immune system [17,18,19]. This type of immunotherapy (IO) is administered using monoclonal antibodies (mAb) that prevent signaling by these molecules and reinvigorate anti-tumor immunity by relieving their suppression of tumor-reactive T cells. ICIs have become a standard of care treatment for various types of NSCLC as well as extensive stage SCLC [20,21]. In NSCLC patients, ICIs are used to treat early- and advanced-stage disease, in first- and second-line settings, as a monotherapy or in combination with chemotherapy and radiotherapy, and as a consolidation therapy after chemoradiation of non-resectable lung tumors [14,20]. Additionally, recently completed and ongoing clinical trials have indicated the benefit of using ICIs in neoadjuvant and adjuvant scenarios [22,23,24,25,26,27,28]. Unlike most lung cancer treatments, ICIs can elicit durable responses of 5 years or more in some patients including those with metastatic disease [29]. The success of PD-1 blockade demonstrates the therapeutic utility of harnessing the immune system to fight cancer, and the promise for additional IO to positively impact lung cancer survival rates.

Numerous IO strategies are being developed and evaluated in clinical trials for the treatment of NSCLC and other malignancies [21,30]. These include but are not limited to oncolytic viruses [31], cancer vaccines [32], and adoptive cell therapies (e.g., CAR-T cells) [33], as well as therapeutic antibodies targeting other checkpoints such as the innate immune checkpoint governed by the CD47–SIRPα axis [21,34]. Signaling through the immunosuppressive molecule, CD47, inhibits the function of phagocytes during innate immunity, thereby diverting anti-tumor immune responses. As such, CD47 blockade is an attractive therapeutic strategy for stimulating tumor immunity. In this review, we describe the tumor immunity cycle and approved ICIs for NSCLC. We then summarize the literature describing CD47′s functions and evidence of the potential of CD47-targeted therapy to become the next IO approved for NSCLC. Finally, we discuss several knowledge gaps with respect to using CD47 blockade effectively in patients and share our perspective on areas of research that should be prioritized to address them.

## 2. Immunotherapy in Non-Small-Cell Lung Cancer

### 2.1. Disruption of the Cancer Immunity Cycle Enables Tumor Immune Evasion

**Figure 1:**
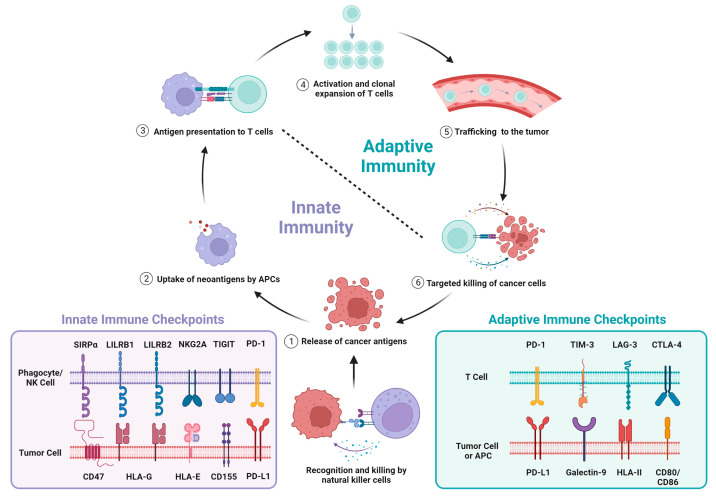
Immune checkpoints govern the cancer immunity cycle. The innate and adaptive immune systems work together in a continuous cycle to recognize and eliminate malignant cells. The cycle is initiated by the death of cancer cells which releases tumor antigens that are recognized as foreign by the immune system. Antigen-presenting cells (APCs), including macrophages and dendritic cells, internalize tumor antigens via phagocytosis, which are processed and presented at the cell surface. APCs travel to the lymph nodes where they present tumor antigens to T cells. When the presented antigen is recognized by its cognate T cell receptor, this triggers T cell activation and clonal expansion of tumor-reactive T cells that recognize cancer-specific antigens. Activated T cells then traffic through the bloodstream to the tumor to carry out tumor-targeted killing. The tumoricidal effects of cytotoxic T cells release more tumor antigens, and the cancer immunity cycle repeats. Natural killer (NK) cells are cytotoxic cells of the innate immune arm that can also kill tumor cells and contribute to neoantigen release. Inhibitory immune checkpoints exist at multiple points throughout the cycle and function to limit immune activation by suppressing key actions like phagocytosis and immune cell-mediated killing of cancer cells.

As described by Chen and Mellman, the cancer immunity cycle is a series of immunological events required to produce an effective anti-tumor immune response to counter neoplastic growth [35] (Figure 1). This cycle is dependent on antigen-presenting cells (APCs), such as macrophages and dendritic cells, to stimulate an initial innate immune response, as well as effector T cells to carry out tumor-targeted killing and adaptive immunity. This imposes negative selective pressure on cancer cells which react by manipulating themselves or stromal cells in the tumor microenvironment (TME) to disrupt the cycle and enable their survival. Tumor immune evasion is a hallmark of many malignancies including lung cancer and is achieved by deregulating the cancer immunity cycle at different stages [36,37,38,39]. In NSCLC, numerous immune escape mechanisms have been reported and are reviewed by Anichini et al. [40]. These include reducing immunogenicity through loss of heterozygosity (LOH) of human leukocyte antigen (HLA), silencing of neoantigens, and impairment of antigen processing and presentation pathways [41,42,43,44,45]. Escape can also be enabled by tumors gaining immunosuppressive capabilities. For example, premalignant lesions and NSCLC can downregulate interferon signaling to avoid tumor-suppressive inflammation, or secrete inhibitory cytokines like IL-10 that remodel the TME to attract tumor-supporting cells like M2-type macrophages and regulatory T cells [45,46,47].

Tumors also co-opt immune checkpoints to facilitate immune escape [47,48,49,50,51,52,53]. Physiologically, a balance of stimulatory and inhibitory signaling is required to maintain immune homeostasis [51,52]. Stimulatory immune checkpoints mount and amplify immune responses, while inhibitory immune checkpoints mediate self-tolerance and prevent autoimmunity and damage to healthy tissues. Tumors often activate inhibitory checkpoints to disarm anti-tumor immune responses [47,48,49,50,51,52,53]. As such, checkpoint molecules are rational therapeutic targets for correcting the cancer immunity cycle and enhancing tumor immunity. Below, we focus on inhibitory checkpoints regulated by PD-1/PD-L1, CTLA-4, and CD47 that suppress innate and adaptive immune cells including APCs and effector T cells that orchestrate tumor immunity (Figure 1).

### 2.2. PD-1/PD-L1 and CTLA-4 Immune Checkpoint Inhibitors (ICIs) in NSCLC

PD-1/PD-L1 and CTLA-4 are inhibitory adaptive immune checkpoint molecules hijacked by many tumor types to suppress T cell-mediated tumor killing [54]. PD-L1 is a ligand recurrently overexpressed on tumor and immune cells that binds to the PD-1 receptor on T cells [55,56]. PD-1/PD-L1 ligation results in T cell exhaustion, allowing tumors to evade destruction by T cell-driven adaptive tumor immunity [20,57]. CTLA-4 is an inhibitory ligand present on T cells that competes with the costimulatory molecule CD28 for binding to B7 receptors (CD80/CD86) on APCs [58,59]. CTLA-4 binding to B7 diminishes APC-mediated T cell priming, activation, and proliferation, thereby inhibiting tumor-targeted adaptive immune responses. After elucidating the roles of these checkpoint molecules in tumor immune escape, therapeutic mAbs were developed to disrupt interactions between PD-1/CTLA-4 and their respective ligands on tumor and immune cells [20,57]. The impact of these discoveries and subsequent translation of ICIs for cancer therapy was recognized by Drs. James Allison and Tasuku Honjo receiving the 2018 Nobel Prize in Physiology or Medicine [60].

**Figure 2:**
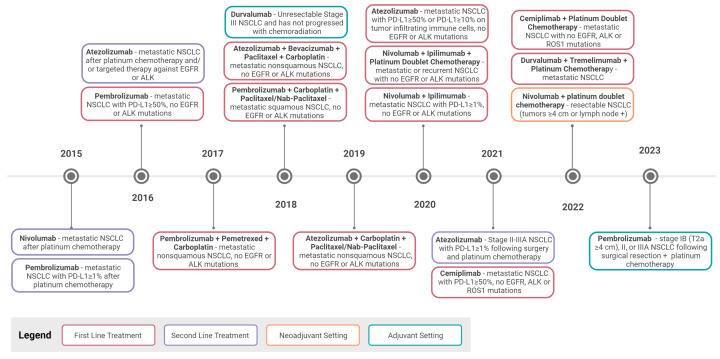
Food and Drug Administration approval timeline for immune checkpoint inhibitors targeting PD-1/PD-L1 and CTLA-4 in non-small cell lung cancer. In 2015 and 2016, the first immune checkpoint inhibitors were approved for use as a second-line treatment in NSCLC patients who progressed on chemotherapy, EGFR- and ALK-targeted therapies. Pembrolizumab became the first IO to be used as a first-line therapy. From 2017 onwards, numerous approvals have been made for using ICIs in a first-line setting for NSCLC either as a monotherapy or combined with chemotherapy or other approved ICIs. IO for NSCLC patients is assigned based on tumor histology, mutation status, and PD-L1 expression levels. ICIs are now also used in adjuvant and neoadjuvant settings. All information was obtained from FDA.gov, accessed on 16 October 2023.

The first clinically translated ICI was Ipilimumab, an anti-CTLA-4 mAb, approved for melanoma in 2011. Following its approval, the FDA-approved anti-PD-1 (Nivolumab and Pembrolizumab) and anti-PD-L1 (Atezolizumab) ICIs in 2014 and 2015 after positive trials in advanced-stage melanoma, renal cell carcinoma, and NSCLC patients [61,62] (Figure 2). Since then, numerous ICIs and combinations have been approved for treating NSCLC, beginning with approvals of Nivolumab and Pembrolizumab (2015), Atezolizumab (2016) [63], and Durvalumab (2018) [17,18,64,65,66] (Figure 2). Patients treated with these antibodies exhibited prolonged progression-free and overall survival compared to chemotherapy-treated individuals [20,67,68,69,70,71]. In 2020, Ipilimumab was approved for treating NSCLC in combination with Nivolumab, marking the first ICI combination approved for NSCLC, which was then approved for combination with chemotherapy [72,73,74]. Collectively, clinical trials have shown that ICIs elicit responses in 20–30% of eligible advanced-stage NSCLC patients and can nearly double 5-year survival rates compared to chemotherapy treatment [75,76], a remarkable feat for patients with metastatic disease.

### 2.3. Limitations of Current Immune Checkpoint Inhibitors

Despite their success in some NSCLC patients, ICI efficacy is limited by imperfect response-predictive biomarkers, restricted applicability, and resistance to therapy. Currently, biomarkers used to assign ICI therapy include tumor driver gene status, PD-L1 expression, and tumor mutation burden (TMB), but they are far from perfect [21,77,78,79,80]. Only 15–20% of PD-L1-positive NSCLC tumors benefit from ICI therapy and some patients with PD-L1-negative tumors also respond [20,62]. Using these biomarkers and other clinical indications for prescribing ICI therapy, only ~15% of NSCLC patients are eligible to receive it [81,82], highlighting the need for new IO options for ineligible patients. Similar to targeted therapies, ICI resistance is a clinical challenge. EGFR- and ALK-driven tumors are inherently resistant to ICIs [83,84,85], and acquired resistance can be conferred through various mechanisms including loss of neoantigens, antigen presentation, and β2M expression, as well as upregulation of alternative inhibitory checkpoint molecules like TIM-3 and LAG-3 [43,86,87,88]. Although there is room to improve the efficacy and utility of ICIs, their success in a subset of NSCLC patients suggests that the development of additional ICIs holds great promise to benefit those who are ineligible, do not respond, or become resistant to CTLA-4 and PD-1/PD-L1 blockade.

## 3. Innate Immune Checkpoints as Targets for Cancer Immunotherapy

As discussed above, tumors can hijack inhibitory immune checkpoints that suppress innate immunity to facilitate immune escape [50,89,90,91], making them attractive targets for IO. For example, several checkpoints inhibit the cytotoxic activity of NK cells and their ability to kill cancer cells including those governed by TIGIT, NKG2A, and the killer immunoglobulin-like (KIR) family of inhibitory receptors (Figure 1). The relevance of NK cell checkpoints in tumor immune evasion and IO are reviewed elsewhere [92,93]. Innate immune checkpoints that suppress phagocyte function and subsequent antigen presentation to T cells are also exploited by tumors to avoid immune-mediated destruction. For instance, binding between HLA class I on cancer cells and leukocyte immunoglobulin-like receptor 1 (LILRB1) expressed on monocytes, dendritic cells, and tumor-associated macrophages transduces an inhibitory signal that blocks phagocytosis [94,95]. A distinct innate immune checkpoint with a well-established role in suppressing phagocytosis is the CD47–SIRPα signaling axis [34]. Binding of CD47 to the SIRPα receptor expressed on APCs transmits a “don’t eat me” signal to prevent tumor cell engulfment by phagocytes [34,53].

Understanding how innate immune checkpoints contribute to tumor immune evasion informed several IO strategies that are being developed or evaluated in ongoing clinical trials [53]. For example, multiple mAb-targeting inhibitory NK cell checkpoints regulated by KIRs, TIGIT, and NKG2A are being assessed in patients based on preclinical evidence of activity in solid and hematological malignancies [93,96,97,98,99]. Similarly, antibodies targeting LILRB1 and LILRB2 alone or in combination with PD-1 blockade have been shown to reinvigorate tumor immunity and are being assessed in early-phase clinical trials with encouraging initial results [100,101,102]. Another IO strategy on the path to clinical translation involves blocking the CD47–SIRPα checkpoint (CD47 blockade) [34]. In the following section, we describe CD47 and its expression, regulation, and functions in the context of cancer biology.

## 4. Cellular Functions of CD47 and Implications in Tumor Biology

### 4.1. CD47 Is a Ubiquitously Expressed Transmembrane Protein Upregulated in Cancer

**Figure 3:**
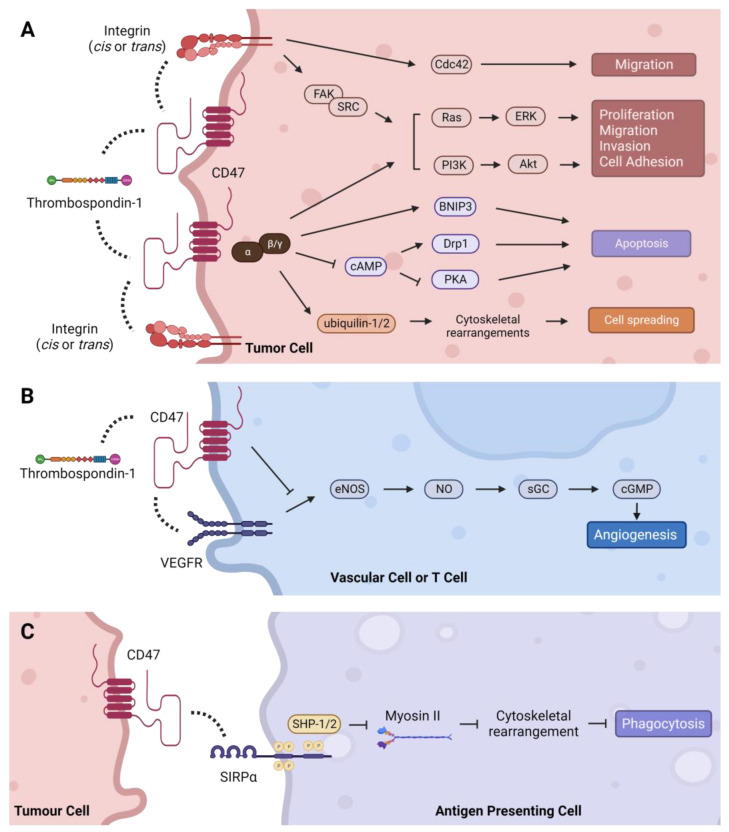
Intracellular signaling pathways regulated by CD47. (**A**) TSP-1 can indirectly activate integrin signaling through CD47 to stimulate Cdc42, as well as the Ras/ERK and PI3K/Akt pathways via FAK/SRC to regulate tumor-promoting processes such as cell proliferation, migration, invasion, and adhesion. CD47 signals through Gi proteins to promote cell proliferation through the PI3K/Akt pathway and cell spreading via direct association with ubiquilins. CD47-Gi interaction also regulates apoptosis by inhibiting cAMP-dependent signaling pathways, and CD47 can induce apoptosis directly through its interaction with BNIP3. (**B**) TSP-1 mediated inhibition of angiogenesis is CD47-dependent. Binding of TSP-1 to CD47 causes CD47 to dissociate from VEGFR2 and inhibits phosphorylation of VEGFR2, thereby suppressing the angiogenesis pathway. (**C**) CD47 binding to SIRPα initiates a signaling cascade that inhibits phagocytosis in macrophages and dendritic cells. Dashed lines indicate protein interactions.

CD47, also known as integrin-associated protein (IAP), is a 50 kDa cell surface protein with an IgV-like extracellular N terminal domain, five transmembrane domains, and a C-terminal cytoplasmic tail with four isoforms [103]. Structurally, the IgV-like extracellular domain is composed of two beta-sheets linked by a cysteine bridge and tethers CD47 to the cell membrane [104,105]. A number of post-translational modifications on CD47 have been identified, including ubiquitination, phosphorylation, and glycosylation, as well as pyroglutamate and heparan sulfate modifications that alter its structure, expression, localization, and function [104,106,107,108,109,110]. The extracellular domain of CD47 binds to multiple ligands, while the cytoplasmic domain associates with Gi and other proteins [111,112]. These interactions transduce signals that regulate a plethora of cellular processes in diverse cell types as summarized below [113,114] (Figure 3).

CD47 is expressed in cells and tissues throughout the body, with relatively high levels on cells derived from the hematopoietic system including erythrocytes (red blood cells, RBCs) and hematopoietic stem and progenitor cells [91,103,115,116,117,118,119]. CD47 is also expressed as a >250 kDa proteoglycan on T cells, endothelial cells, and vascular smooth muscle cells [106]. Oldenborg and colleagues reported CD47 as a “marker of self” after discovering that RBCs lacking CD47 were eliminated by splenic macrophages [116]. Subsequent studies found that CD47 expression on RBCs decreases over their lifetime to mark aged cells for phagocytic clearance [115,120]. Regulation of platelet homeostasis via CD47 expression has also been described [121]. Upon recognition of its role in inhibiting phagocytosis, akin to CD31 disabling phagocytosis of viable cells, CD47 was later coined a “don’t eat me” signal [122]. CD47 is upregulated in many malignancies, and the first observations of cancer-associated CD47 overexpression were made in ovarian cancer [123,124]. Since then, CD47 expression has been described in various tumor types including NSCLC and SCLC, with numerous studies reporting its association with tumor stage and patient survival (Table 1).

### 4.2. Regulation of CD47 Expression in Cancer

Several mechanisms have been described to regulate CD47 transcription in cancer cells [155]. Expression of CD47 can be controlled by cytokines, oncogenes, and micro-RNAs (miRNA). In liver and breast cancer cells, TNFα induces CD47 expression through the transcriptional regulator, NF-κB [156,157]. In melanoma models, CD47 expression was induced by IFNγ, although the exact mechanism remains to be elucidated [158,159]. Interleukins also appear to upregulate CD47 expression. IL-6 induced CD47 through STAT3 in hepatoma cells, and IL-1β induced CD47 via NF-κB in cervical cancer cells [147,160]. CD47 induction by IL-4, IL-7, and IL-13 has also been described, but how these interleukins stimulate CD47 transcription is unknown [160,161]. These immune-stimulating, pro-inflammatory cytokines may be physiologically programmed to induce CD47 expression, like PD-L1, as a negative feedback mechanism to prevent harmful overactivation of the immune response. Both MYC and HIF-1A oncogenes have been shown to directly bind the CD47 promoter and induce its transcription in breast, leukemia, and lymphoma models [162,163]. On the contrary, multiple miRNA have been shown to negatively regulate CD47 expression in several cancer types by degrading CD47 transcripts or blocking protein translation. These include miR-133a, miR-155, miR-192, miR-200a, miR-222, miR-340, and miR-708 [155]. Two recent studies established novel mechanisms of CD47 expression regulation by the oncogenic drivers, EGFR and KRAS. In NSCLC, KRAS was discovered to upregulate CD47 by suppressing miR-34a [164]. Specifically, KRAS-mediated activation of PI3K-AKT signaling led to the phosphorylation of STAT3 and its transcriptional repression of miR-34a, thereby relieving the post-transcriptional inhibition of CD47 and increasing its expression. Similarly, EGFR was found to upregulate CD47 by stabilizing its expression. In glioblastoma models, EGFR activation induced c-Src-mediated phosphorylation of CD47, which prevented its interaction with the E3 ubiquitin ligase, TRIM21, and protected CD47 from ubiquitin-associated degradation [110]. 

### 4.3. CD47: Molecular Interactions, Signaling Pathways, and Malignant Phenotypes

CD47 regulates various signaling cascades by interacting with different proteins including those located intra- or extracellularly, within the plasma membrane or in the extracellular matrix (ECM). These interactions occur in either a cis (same cell) or trans (different cell) manner and are dependent upon CD47′s IgV-like, transmembrane, and cytoplasmic tail domains. The most well characterized CD47 interaction partners include thrombospondin-1, integrins, and members of the signal-regulatory protein (SIRP) family, which signal through CD47 to promote various hallmarks of cancer (Figure 3). 

#### 4.3.1. Thrombospondin-1 (TSP-1)—Proliferation, Migration, Cell Death, and Angiogenesis

TSP-1 is an extracellular matrix (ECM) glycoprotein secreted by platelets, macrophages, dendritic cells, endothelial cells, smooth muscle cells, and epithelial cells in response to stress, as well as tumor and stromal cells in the TME. TSP-1 interacts with many proteins including integrins, collagen, fibrinogen, laminin, proteases, and growth factors to regulate diverse physiological processes such as vascular response to injury, inflammation, platelet activation, and ECM remodeling [165,166]. The cellular effects of TSP-1 are dependent on tissue-specific expression of its receptors and other interacting partners in the local environment. The C-terminal domain of TSP-1 binds the extracellular region of CD47 at picomolar concentrations to control motility, proliferation, and angiogenesis that influence the invasive and metastatic properties of cancer cells [165]. For example, CD47–TSP-1 interaction promoted tumor progression in a T cell lymphoma model by supporting proliferation, survival, and migration via activation of ERK, AKT and survivin signaling [167] (Figure 3A). Similarly, antibody-mediated cross-linking of CD47 on T cells stimulates their activation and proliferation [168,169]. In contrast, TSP-1-induced cell death in leukemia, lung, breast, and colon cancer cell lines in a CD47-dependent, caspase-independent manner [170,171,172]. In non-malignant T cells, binding of CD47 by TSP-1 has been shown to induce caspase-independent apoptosis and stimulate migration, and to suppress activation-induced proliferation, CD69 expression, and IL-2 production, further illustrating the context-specific effects of CD47–TSP-1 interactions [106,173,174,175,176]. CD47–TSP-1 binding has also been implicated in mediating sensitivity to cancer therapies. Interaction of CD47 and TSP-1 blocked the escape of breast and colorectal cancer cells from chemotherapy-induced senescence and sensitized melanoma cells to radiotherapy in a cell autonomous manner [177,178]. CD47 blockade also protected mice from lethal whole-body irradiation that was associated with an increase in autophagy in surviving cells [179]. However, the specific mechanisms explaining these responses to therapy remain to be defined. 

Studies in endothelial cell models have deduced a role for CD47 in regulating angiogenesis through the VEGFR2 signaling pathway [113,180] (Figure 3B). First, CD47 was found to be essential for TSP-1-mediated inhibition of nitric oxide-stimulated responses in vascular cells [181]. It was later discovered that CD47 physically associates with VEGFR2 in endothelial and T cells, and that CD47 ligation by TSP-1 disrupts this physical interaction with VEGFR2, which inhibits its phosphorylation and downstream signaling [182,183]. It is unclear how the CD47–TSP-1–VEGFR2 axis influences angiogenesis in tumors because of conflicting findings in tumor models, which may be confounded by irregularities in tumor vasculature and the enigmatic role of nitric oxide in regulating tumor angiogenesis [184]. Studies in melanoma and breast tumors found that TSP-1 overexpression in cancer cells negatively regulated tumor blood flow in response to vasoactive agents in a CD47-dependent manner [185]. Recent studies reported that disrupting the CD47–TSP-1 interaction reduced angiogenesis in neuroblastoma and glioblastoma models, but selectively depleting CD47 in stromal cells increased angiogenesis and tumor burden in a syngeneic prostate cancer model [186,187]. Furthermore, inhibition of CD47 normalized tumor vasculature in a multiple myeloma model, which was associated with reduced expression of pro-angiogenic factors, increased expression of anti-angiogenic factors, and tumor growth inhibition [188]. Thus, the effects of TSP-1-CD47 interactions on angiogenesis may be malignancy-dependent, and further studies are needed to fully understand them. 

#### 4.3.2. Integrins—Migration, Invasion, and Inflammation

CD47 was originally named integrin-associated protein (IAP) because it was discovered that it binds with several members of the integrin family of transmembrane receptors, including integrins αvβ3, αIIbβ3, and αβ1 [112]. Subsequently, CD47 has been shown to interact with α5, α4β1, α6β1, among others [180]. Integrins facilitate cellular attachment to the ECM via the actin cytoskeleton and regulate focal adhesion kinase (FAK), integrin-linked kinase (ILK), and SRC kinase signaling pathways including Ras-ERK, PI3K/AKT, and YAP/TAZ, which support tumor growth and progression [189] (Figure 3A). Lateral (cis) interactions between CD47 and integrins form signaling complexes that can activate integrins and influence binding to ECM proteins [114,180]. They also promote migration and metastasis phenotypes in a seemingly cancer-specific manner [114], which may be explained by differences in integrin expression across cancer types. Interactions between CD47 and αvβ3 enhanced binding of ovarian and breast cancer cells and spreading of melanoma cells on vitronectin-coated substrates, as well as chemotaxis of prostate cancer and melanoma cells towards collagen [190,191,192,193]. CD47-α4β1 interactions stimulated adhesion in melanoma, lymphoma, and T cells, and promoted migration in B-cell leukemia models [194,195,196]. Notably, many of these processes are dependent on the ligation of CD47 by TSP-1, TSP-1-derived peptides (e.g., 4N1K), or anti-CD47 antibodies. In addition to these cancer cell-intrinsic effects, CD47–integrin interactions can influence tumor immunity by modifying the behavior of immune cells in the TME [112]. For instance, CD47-blocking antibodies inhibited CD23-stimulated secretion of several pro-inflammatory cytokines including TNFα, IL-1β, and PGE2 from monocytes and IFNγ from T cells through a CD47–αvβ3 complex [197]. Consistently, CD47 ligation with antibodies or 4N1K suppressed IL-12 release from monocytes and inhibited the transition of naive T cells to Th1 effector cells [198,199]. Additional mechanistic studies to investigate CD47–integrin interactions in different cancer models are needed to better understand the generalizability of their cellular effects. 

#### 4.3.3. SIRPα/γ—Phagocytosis and Tumor Immune Evasion

The SIRP family is a group of cell surface receptors primarily expressed on myeloid cells [200]. Of the three identified members, SIRPα binds CD47 with the highest affinity and is expressed on macrophages, dendritic cells, T cells, hematopoietic progenitor cells, and neurons [111]. Hatherley et al. identified five key residues (Y37D, D46K, E97K, E100K, E106K) in the extracellular domain of human CD47 that are critical for binding to SIRPα [104]. In addition, Gln-19 must be enzymatically modified to pyrrolidone carboxylic acid (pyroglutamate) for SIRPα to bind CD47 [108]. CD47–SIRPα interaction initiates a signaling cascade that inhibits phagocytosis, and this pathway has been the primary focus for developing CD47-targeted therapies (Figure 3C). Upon CD47–SIRPα ligation, the intracellular immunoreceptor tyrosine-based inhibitory motif (ITIM)-domain in the cytoplasmic tail of SIRPα is phosphorylated. This recruits the inhibitory phosphatases, SHP-1 and SHP-2, which dephosphorylate myosin II, thereby preventing reorganization of the cytoskeleton that is required for phagocytosis to occur [201]. Consequently, antigen uptake and presentation by APCs and subsequent activation of the adaptive immune response is inhibited [202]. In addition to SIRPα, SIRPγ also binds to CD47 but with ten times lower affinity [203]. SIRPγ is expressed on NK cells and lymphocytes, and studies have shown that CD47–SIRPγ interactions positively regulate T cell transendothelial migration, T cell activation, and apoptosis of Jurkat cells, which could be undesirably impaired by CD47 blockade [203,204,205,206].

#### 4.3.4. Intracellular Interactions and Signaling

Many of the interactions described above transduce extracellular signals to intracellular molecules bound to CD47′s cytoplasmic tail to elicit associated cellular effects (Figure 3A,B). Initially, CD47 was co-immunoprecipitated with heterotrimeric Gi proteins in membranes isolated from platelets, melanoma, and ovarian cancer cells, which was reversible by the potent inhibitor of receptor–Gi protein binding, pertussis toxin [207]. Evidence that CD47 activates Gi proteins was provided by the finding that 4N1K ligation decreased intracellular cAMP levels [207]. Later studies found that CD47 interacts with two ubiquitin-related proteins involved in protein degradation, ubiquilin-1 and ubiquilin 2, via tethering to CD47 by Gβγ [208,209,210]. This interaction induced cytoskeletal rearrangements and enhanced spreading of Jurkat and ovarian cancer cells [209]. Subsequently, the CD47–Giαβγ pathway was shown to activate PI3K/Akt signaling to induce proliferation in astrocytoma cells [211]. Additional cytoplasmic signaling cascades regulated by TSP-1–CD47-mediated Gi activation and consequential reductions in cAMP levels include the phosphorylation of SYK and LYN and their interaction with FAK during platelet aggregation; phosphorylation of ERK during smooth muscle cell migration and T cell lymphoma migration and adhesion; and inhibition of PKA during apoptosis in activated T cells [167,195,212,213,214]. In addition to Gi proteins, the cytoplasmic domain of CD47 has also been shown to directly interact with the BCL-2 family member, BNIP3, to induce a necrosis-like mode of caspase-independent T cell death upon CD47 ligation with TSP-1 [175]. Most recently, c-Src was reported to bind and phosphorylate CD47 in an EGFR-dependent manner, which led to CD47 stabilization and immune evasion in a glioblastoma model [110]. Besides these direct physical associations, CD47 has been discovered to indirectly interact with several other cytoplasmic proteins to regulate various cancer-relevant processes. These include interactions with Cdc42, Drp1, and guanylate cyclase (GC), as well as regulation of autophagy proteins that collectively influence cell death and survival, migration, invasion, and angiogenesis [113,179,180,215,216]. 

## 5. CD47 Is a Clinically Relevant Therapeutic Vulnerability in Non-Small Cell Lung Cancer

The remainder of this review summarizes evidence of the clinical relevance and therapeutic potential of CD47 in NSCLC, strategies for achieving CD47 blockade that are currently being developed and evaluated in clinical trials, and key challenges associated with translating CD47-targeted therapy into oncology clinics. Below, we report the findings of studies identified using the PubMed search “lung AND (cancer OR tumor OR tumour) and (CD47 or SIRPα)”.

### 5.1. CD47 Expression and Clinical Significance in NSCLC

Overexpression of CD47 mRNA in lung cancer relative to non-malignant tissues was first discovered in SCLC cell lines and patient-derived xenografts [217]. Weiskopf and colleagues later confirmed that CD47 was upregulated in SCLC and reported CD47 to be a promising immunotherapeutic target, which stimulated numerous investigations into CD47′s clinical significance in lung cancer [218]. Since then, several transcriptomic and proteomic studies have demonstrated CD47 expression in SCLC, LUAD, and LUSC tumors and CD133+ lung cancer stem cells [126,131,219,220,221]. Data from published reports that measured CD47 expression using immunohistochemistry (IHC) suggest that CD47 is expressed in 28–85% of SCLC and 30–84% of NSCLC. Although these percentages are variable, they consistently indicate that CD47 expression is a recurrent feature in lung cancer regardless of subtype (Table 1). Moreover, high mRNA and protein expression of CD47 was associated with advanced tumor stage, metastasis, recurrence, and/or survival in the majority of NSCLC and SCLC studies that we assessed (Table 1). Additionally, multiple studies have identified higher CD47 expression in LUAD compared to LUSC [125,219].

Besides analyzing associations between CD47 and prognostic features, multiple groups have assessed how CD47 protein expression correlates with immune cell infiltration and PD-L1 expression. One study of 430 NSCLC patients found that PD-L1 and CD47 were co-expressed in 23.7% of LUSC and 14.6% of LUAD [131]. Moreover, co-expression was associated with increased CD8 T cell infiltration in both subtypes and with high macrophage (CD68+) density in LUSC, although additional markers to infer T cell activation versus exhaustion and macrophage functionality (i.e., M1 versus M2) were not assessed [131]. Co-expression of PD-L1 and CD47 was found to be an independent prognostic indicator of poor survival in both LUAD and LUSC patients, suggesting they may cooperate to suppress tumor immunity [131]. A positive association between CD47 expression and CD68+ macrophages was also found in a study of 384 NSCLC specimens [127]. Another study of 191 NSCLC found no correlation between CD47 and M1 (Cd68+Cd163-) or M2 (Cd68+Cd163+) macrophage or CD8 T cell infiltration, but identified a significant negative correlation between CD47 and PD-L1 expression [125]. A similar trend towards a lower frequency of PD-L1 positivity in CD47-high tumors was seen in a study of 169 NSCLC tissues [50]. Lastly, an assessment of a NSCLC cohort with 98 samples revealed that CD47 expression on tumor cells was positively correlated with FOXP3+ T cells, but was not correlated with PD-L1 or CD68+ macrophage markers [129].

*EGFR* and *KRAS* are the two most commonly mutated oncogenes in LUAD. Multiple studies have shown that *EGFR*-mutant tumors are enriched for high CD47 expression at both the protein and mRNA levels [50,125,127,222]. CD47 expression levels are also higher in LUAD patients with no smoking history compared to smokers, consistent with the higher frequency of *EGFR* mutations in never-smoker lung cancer [125,221]. A single study found that CD47 expression was elevated in *KRAS* mutant compared to *KRAS* wildtype tumors in three independent LUAD cohorts, but *EGFR* was not considered in the analyses [164]. Supporting the positive associations between CD47 expression and these oncogenes, recent reports identified mechanisms whereby EGFR and KRAS upregulate CD47 expression in cancer cells as described above [110,164]. 

Differences in the findings across these correlative NSCLC studies may reflect disparate patient or tumor characteristics in the individual cohorts, antibody-staining conditions, or differences in thresholds used for scoring CD47 positivity. Thus, further studies in sufficiently large NSCLC cohorts using standardized IHC protocols are required to corroborate the correlations identified to date, as some of them could be useful biomarkers for prescribing CD47 blockade. For example, PD-L1 expression could confer resistance to CD47 inhibition, while *EGFR* and *KRAS* mutations could indicate sensitivity. Clinical trials in NSCLC patients will determine the validity of these hypotheses. Some of the CD47 correlations identified, such as those between CD47 expression and *EGFR* mutation, lung cancer stage, and metastasis (see Table 1) can be explained by underlying regulatory mechanisms (i.e., EGFR upregulates CD47 expression as described in Section 4.2., and CD47 promotes lung cancer migration and metastasis, see Section 5.3.2.). However, additional functional studies are required to explore the mechanisms underlying other putative associations, such as the reported negative correlation between CD47 and PD-L1 expression.

### 5.2. Pathways to CD47 Upregulation in NSCLC

EGFR and KRAS are abnormally activated by DNA amplification and/or mutations in approximately 15% and 30% of NSCLC, respectively [223], and their overactivation was recently identified to upregulate CD47 expression. Although the EGFR-CD47 axis was primarily defined using glioblastoma models, it likely operates in NSCLC as EGF stimulation induced CD47 expression in A549 cells and CD47 positivity is associated with *EGFR*-mutant LUAD [50,110,125]. Conversely, the KRAS–STAT3–miR34a mechanism was described specifically in LUAD but likely applies to other *KRAS*-driven tumors [164]. *MYC* is also recurrently amplified and overexpressed in NSCLC and CD47 and is a direct target of MYC-driven transcription [162,224,225]. Given that CD47 is expressed in up to 84% of NSCLC, oncogenes are unlikely to account for CD47 upregulation in all cases. Studies conducted in various models have established that IFNγ regulates CD47 expression in NSCLC [130,226]. IFNγ is a proinflammatory cytokine that can directly induce apoptosis in tumor cells and stimulate innate and adaptive immune activation. On the other hand, IFNγ can promote metastasis and immune escape by upregulating immune checkpoint molecules like PD-L1 and IDO [227]. Ye and colleagues first described induction of CD47 by IFNγ in lung cancer cells through a JAK–STAT1–IRF1 signaling pathway [226]. Later, this mechanism was confirmed by Qu et al. who demonstrated that IRF1 binds to the CD47 promoter and induces its transcription [130,226].

In addition to oncogenes and cytokines, multiple groups have shown that standard of care lung cancer therapies upregulate CD47 expression [130,226,228]. In vitro studies in mouse and human NSCLC models found that cisplatin induces CD47 expression [228]. Another study showed that radiotherapy enhances expression of CD47 through the JAK2–STAT3 pathway in a murine model of NSCLC [229]. Concordantly, cisplatin has been shown to induce IRF1 activation, and irradiation has been shown to induce type I (IFNα and IFNβ) and type II (IFNγ) interferons in cancer cells [230,231,232,233,234]. Thus, upregulation of CD47 by these therapies could be attributable to therapy-induced DNA damage and cytoplasmic DNA that stimulates the cGAS–STING pathway [235,236,237], leading to interferon induction and activation of JAK/STAT-driven transcription and signaling. Furthermore, expression of CD47 and IFNγ was elevated in lung tumor tissues treated with the multi-kinase inhibitor, sorafenib [130]. Taken together, this evidence suggests that multiple lung cancer therapies could upregulate CD47 through a JAK–STAT–IFN pathway. Notably, treatment of lung cancer cells with the EGFR tyrosine kinase inhibitor, gefitinib, or the KRAS-G12C inhibitor, AMG 510, reduced CD47 expression, which is consistent with the newly discovered mechanisms of CD47 regulation by EGFR and KRAS [110,164,222]. 

### 5.3. CD47 Promotes NSCLC Growth and Progression

Studies in preclinical NSCLC models have demonstrated that inhibition of CD47 using genetic or pharmacologic approaches impairs malignant phenotypes including tumor growth and immune escape, metastasis, stemness, and drug resistance. Below, we review the functional studies providing evidence to support these tumorigenic roles of CD47 in NSCLC. These findings reinforce the therapeutic potential of CD47-targeted therapy in NSCLC.

#### 5.3.1. Tumor Growth

The first report demonstrating that disrupting the CD47–SIRPα interaction in lung cancer had anti-tumor effects was that by Weiskopf and colleagues, who showed that CD47 blockade with a mAb in SCLC cell lines and patient-derived xenografts enhanced cancer cell phagocytosis by macrophages [218]. They also showed that treatment with CD47 mAb impaired tumor growth and prolonged the survival of NSG mice bearing human H82 cell line xenografts, and that genetic ablation of CD47 in the KP1 model impaired tumor growth in immunodeficient and immunocompetent mice. Although this study investigated SCLC, a number of similar studies in NSCLC were published shortly thereafter. Knockdown of CD47 using shRNA or siRNA in A549 cells or tumor xenografts, as well as shRNA in a Kras–Trp53-driven genetically engineered mouse model of LUAD, was found to increase phagocytosis of lung cancer cells by macrophages, reduce tumor growth, and prolong the survival of tumor-bearing mice [126,164,238]. Antibody-mediated CD47 blockade also enhanced phagocytosis by macrophages and dendritic cells and inhibited tumor growth in A549 and H1975 xenograft as well as LLC syngeneic tumor models, which was associated with an increase in intratumoral macrophages [219,222,239,240]. Taken together, these independent studies indicate that CD47 blockade suppresses lung tumor growth by inducing phagocytic clearance of cancer cells by DCs and macrophages, which can engage adaptive immune responses to further inhibit tumor progression in immunocompetent hosts. Additional studies are needed to determine (i) whether phagocytosis induced by CD47 blockade in lung cancer is differentially executed by M1 versus M2 macrophages as previously suggested [34], and (ii) whether immune-independent effects of CD47 inhibition also contribute to its anti-tumor efficacy. Nevertheless, the data to date suggest that inhibiting CD47 is a promising therapeutic strategy for NSCLC.

#### 5.3.2. Migration and Metastasis

Other studies have implicated a cell-intrinsic role for CD47 in facilitating NSCLC cell migration and metastasis. Functional studies in A549 demonstrated that CD47 promotes invasion and migration in vitro as well as tumor growth and metastasis in vivo [126]. Mechanistic investigation uncovered that CD47 regulates the Rho family small GTPase, Cdc42, to promote actin reorganization that is required to drive cell motility and invasion and subsequent metastasis. This hypothesis was supported by the finding that Cdc42 knockdown abrogated metastasis induced by CD47 overexpression [126]. More recently, an analysis of 120 LUAD tissues found that CD47 expression was higher in advanced-stage tumors, suggesting an association with metastasis that was linked to upregulation of IFNγ in the TME [130]. Indeed, CD47 KO in A549 cells attenuated IFNγ-stimulated invasion and migration in vitro and tumor metastasis in mice [126,130]. Although the specific mechanism underlying CD47′s contribution to tumor metastasis remains to be defined, these studies suggest that CD47 may also contribute to tumor progression by supporting metastatic dissemination.

#### 5.3.3. Cancer Stem Cells

Liu et al. explored CD47 expression specifically on CD133+ lung cancer stem cells (LCSC) [219]. Using flow cytometry on dissociated human tumor and non-malignant lung tissues, they confirmed that CD47 expression was higher in tumors compared to normal cells, but also discovered that CD133+ LCSC had the highest expression levels overall. Functionally, CD47 blockade using anti-CD47 or anti-SIRPα mAb significantly increased macrophage phagocytosis of LCSC isolated from NSCLC cell lines and patient tumors. A second study that investigated immunomodulatory molecules in LCSC obtained from lymph node aspirates, confirmed that CD47 expression was higher on cancer stem cells compared to mature tumor cells [241]. These findings may suggest that CD47 has a role in maintaining the stem cell compartment in NSCLC.

#### 5.3.4. Therapy Resistance

Lung cancer patients often experience disease relapse due to acquired drug resistance that can arise through many different mechanisms [10,242,243]. In this regard, some studies have identified associations between CD47 and NSCLC therapy resistance. CD47 levels were found to be higher in LUAD tissues following treatment with sorafenib compared to tumor tissues prior to treatment, consistent with a report that CD47 was upregulated in liver cancers resistant to sorafenib [130,157]. Similarly, although the EGFR TKI gefitinib downregulated CD47 expression in treatment-naive NSCLC cells, CD47 was upregulated in cells with acquired TKI resistance [222]. Moreover, Zhang et al. described elevated CD47 expression in multiple NSCLC lines refractory to treatment with anti-angiogenic VEGF inhibitors, which was induced by TNFα/NF-κB signaling. They further showed that combining SIRPα–Fc antibodies with VEGF inhibition potentiated anti-tumor efficacy by increasing macrophage-mediated phagocytic uptake of cancer cells and inhibiting the formation of new blood vessels in tumors [239]. Finally, CD47 may also influence therapy resistance through its regulation of autophagy [114]. Multiple studies have indicated that CD47 deficiency activates autophagy to promote cell and tissue survival following irradiation [179,215]. In NSCLC cell and xenograft models, SIRPαD1-Fc treatment stimulated autophagic flux, and combining CD47 blockade with autophagy inhibitors enhanced cancer cell killing through a mechanism dependent on ATG5, ATG7, and Beclin 1 [240]. Additional functional and mechanistic studies are required to understand whether CD47 directly supports lung cancer therapy resistance, but these reports rationalize further investigation since CD47 blockade could be a relevant line of treatment for patients resistant to specific NSCLC treatment modalities. 

## 6. CD47-Targeted Immunotherapies for Cancer

### 6.1. Assessment of CD47 Blockade in Preclinical Models

Preclinical work has demonstrated encouraging anti-cancer effects of CD47-targeted inhibition in hematological and solid tumor xenograft and syngeneic models [218,237,244,245]. Strategies for antagonizing CD47 in cancer models have primarily involved the use of anti-CD47 mAbs delivered via intraperitoneal injection, intratumoral injection, or ex vivo coating of tumor cells prior to implantation into mice [158,219,228,237,244,246]. Studies in syngeneic cancer models have shown that CD47 mAbs can effectively inhibit tumor growth and that efficacy is dependent on macrophages, dendritic cells, T cells, and their interactions [237,244,246,247]. As such, most mechanistic investigations of CD47 blockade have focused on characterizing phagocytosis and adaptive tumor immunity that follows. However, other immunologic responses such as antibody-dependent cellular cytotoxicity/phagocytosis (ADCC/ADCP), complement- dependent cytotoxicity (CDC), and cell-intrinsic effects of CD47 ligation on apoptosis, proliferation, and migration could also contribute to anti-tumor efficacy in specific contexts. In fact, some therapeutics and combination strategies have been purposefully designed to stimulate ADCC/ADCP to potentiate the effects of CD47 blockade as discussed below. 

The efficacy of CD47 blockade has also been demonstrated in human tumor xenografts. However, caution must be taken when interpreting these studies since xenografts are grown in immune-deficient hosts such as NOD/SCID mice that have defective NK cells and lack B and T cells. Such models preclude interactions between innate and adaptive immune cells that contribute to response to CD47 blockade. Results derived from tumor studies in the NOD strain of mice are further complicated by the fact that human CD47 binds to NOD-Sirpα with 10× greater affinity than to human SIRPα [248]. Moreover, humanized anti-CD47 therapeutic antibodies used to treat human tumor xenografts do not recognize CD47 on mouse cells, which would otherwise be bound by CD47 mAbs due to its ubiquitous expression. Thus, the “antigen sink” effect is not accounted for when using human-specific antibodies to treat xenografts in mice. Despite these limitations, the inhibition of CD47 in human tumor xenografts has proven to be effective, impairing the growth of lymphoma, leukemia, glioblastoma, breast, colon, ovarian, and lung cancer models [244,249]. Collectively, preclinical studies of CD47 blockade have demonstrated encouraging activity associated with enhanced phagocytic clearance of cancer cells and induction of anti-tumor immunity, stimulating the development of numerous agents for CD47-targeted therapy.

### 6.2. Clinical Strategies for Augmenting Tumor Immunity with CD47 Blockade

**Figure 4:**
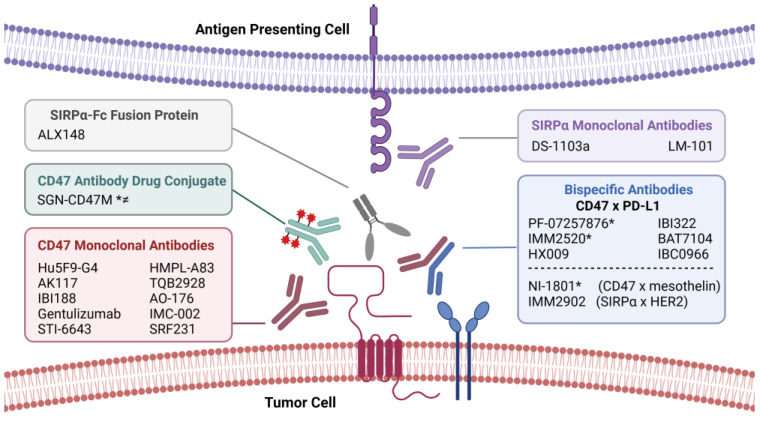
CD47-targeted therapies being investigated in clinical trials including NSCLC patients. There are 22 immune checkpoint inhibitors targeting the CD47–SIRPα axis being evaluated in clinical trials with NSCLC or solid tumor patients: ten CD47 mAb, one CD47 antibody–drug conjugate, eight CD47 bispecific antibodies, one SIRPα fusion protein, and two SIRPα mAb. All information was obtained from the National Institutes of Health registry (clinicaltrials.gov, accessed on 16 October 2023). * Asterisks indicate agents being evaluated specifically in NSCLC patients. All other agents are being tested in patients with “solid” or “malignant” tumors. ≠ Clinical trial terminated.

Several strategies to inhibit the CD47–SIRPα checkpoint have been evaluated in clinical trials for cancer IO (Figure 4; Table 2). These include monoclonal and bispecific antibodies targeting CD47 or SIRPα, as well as soluble SIRPα–Fc fusion proteins. These agents are designed to block the interaction between CD47 and SIRPα to relieve suppression of cancer cell phagocytosis by APCs in order to stimulate tumor immunity. The progress of these agents towards clinical translation is outlined in several recent reviews [250,251,252], and many of them are being tested in combination with other treatments such as IO, chemotherapy, or targeted therapy [253,254,255,256]. According to the National Institutes of Health registry, clinical trials evaluating at least 12 anti-CD47 and 2 anti-SIRPα mAbs are underway in patients with advanced solid or hematologic cancers [251]. In addition to inhibiting binding between CD47 and SIRPα, antibodies targeting CD47 have the potential added benefit of blocking tumor-promoting cell-intrinsic CD47 signaling, but at the cost of challenges associated with the CD47 antigen sink. Most of these therapeutic mAbs contain the IgG4 heavy-chain isotype that has a relatively weak affinity for Fcγ receptors compared to IgG1, a potent inducer of ADCC/ADCP. Thus, CD47 inhibition and Fc-mediated immune effector functions may contribute to the anti-tumor efficacy of IgG1-based anti-CD47 antibodies [257]. 

Bispecific antibodies have been engineered with one Fab fragment targeting CD47 and the other with high affinity for a tumor-specific antigen, enabling them to recognize two different molecules simultaneously. This design was supported by studies that identified synergy when CD47 and PD-L1 antibodies were combined, and others that combined CD47 mAbs with antibodies targeting tumor-specific markers such as CD20 in lymphoma models [258]. In theory, bispecifics should focus the therapeutic effects of CD47 blockade more specifically on tumor cells while minimizing adverse effects that result from ubiquitous CD47 expression. At present, there are eight bispecific Abs under investigation in clinical trials enrolling NSCLC patients, including those targeting CD47 or SIRPα along with PD-L1, mesothelin, and HER2 (Figure 4). Another approach for minimizing antigen sink-related toxicities involves the use of SIRPα–Fc fusion proteins which are engineered antibody constructs consisting of IgG Fc domains fused to specific CD47-binding domains of SIRPα. Soluble SIRPα–Fc fusion proteins function as decoys by competing with cellular SIRPα for binding to CD47. They enhance phagocytosis on their own and in combination with other therapeutic antibodies, and have been shown to minimize RBC toxicity [259,260].

In addition to these clinic-ready therapeutics, several other strategies for achieving CD47 blockade are in earlier stages of preclinical development. These include CD47-targeting siRNA and miRNA (e.g., miR-155) to downregulate CD47 expression [261,262]; TSP-1 derived CD47 agonists to induce CD47-dependent cell death (e.g., PKHB1 and 4N1K) [214,263]; AUR103 and other small molecule inhibitors of QPCTL that prevent the addition of pyroglutamate on CD47, which is required for SIRPα binding [108,264]; and antibody–drug conjugates that combine anti-CD47 antibodies with the drugs, mertansine and VCMMAE [265,266].

Recently, Son and colleagues conducted a meta-analysis of 24 completed clinical trials that evaluated seven CD47 mAbs, six SIRPα–Fc fusion proteins, and one SIRPα mAb alone or in combination with ICIs, targeted therapy or chemotherapy [267]. Their analysis of 771 evaluable hematologic or solid cancer patients revealed that 6.4% had a complete response, 10.4% had a partial response, and 26.1% had stable disease, amounting to an overall objective response rate (ORR) of 16.7%, a disease control rate of 42.8%, and a 4.8 month response duration. Notably, ORR was higher in hematologic versus solid cancers (25.3% versus 9.1%). In solid tumors specifically, SIRPα antagonists, both as monotherapy (16.2% versus 2.8%) and in combination (28.3% versus 3%), yielded higher ORRs than CD47 mAbs, with responses observed in head and neck, colorectal, endometrial, ovarian, liver, NSCLC, and gastric cancer patients. Grade 3–4 treatment-related adverse events occurred in 18% of patients, with the most common being thrombocytopenia, neutropenia, and anemia, consistent with high levels of CD47 expression on platelets, neutrophils, and RBCs, respectively. Overall, these early trial results indicate that CD47/SIRPα blockade was well tolerated and elicited anti-tumor activity in multiple malignancies. Additional trial data that becomes available will inform more effective strategies for using CD47 blockade in specific cancer types, and therapeutic combinations to maximize therapeutic benefit.

It is important to note that several clinical trials testing CD47 blockade have been terminated for administrative reasons, with some discontinued due to lack of efficacy. For example, the NCT02641002 trial testing TTI-621 (CC-90002) monotherapy in acute myeloid leukemia (AML) and high-risk myelodysplastic syndrome (MDS) was terminated because of the poor therapeutic profile. However, this SIRPα–Fc fusion protein is still under investigation as a combination therapy with doxorubicin in leiomyosarcoma (NCT04996004) and with pembrolizumab in diffuse large B-cell lymphoma (NCT05507541). Moreover, two phase 3 trials evaluating combinations of the most advanced CD47 mAb, Hu5F9-G4 (Magrolimab), with azacitidine in AML patients with *TP53* mutations and in MDS patients (NCT04778397 and NCT04313881, respectively) were recently terminated due to failure to yield survival benefits. Discontinuation of these trials testing CD47 blockade as a monotherapy and in combination with other drugs emphasizes the need for preclinical studies to define rational and effective combinations as discussed in detail in Section 7.4 below. 

## 7. Challenges to Overcome for the Success of CD47 Blockade in Cancer

After nearly one decade of using PD-1/PD-L1 and CTLA-4 ICIs as standard of care in oncology clinics, it is clear that the success of IO depends on a strong understanding of the mechanisms governing therapeutic effects, robust response-predictive biomarkers, strategies to mitigate potential toxicities, and combination approaches to maximize efficacy. Below, we summarize outstanding questions that must be addressed for CD47-targeted therapy to become the next successful IO for NSCLC and other cancers.

### 7.1. Mechanisms Contributing to the Anti-Tumor Effects of CD47 Blockade: How DOES It Work? 

Most studies investigating CD47 blockade in tumor models have attributed its anti-tumor effects to enhanced phagocytosis and adaptive tumor immunity mediated by APCs and CD8 T cells [237,244,246,247]. However, neutrophils and NK cells may also be involved [268,269], and CD47 mAbs can positively or negatively regulate T cell function [113,114]. Tumor growth inhibition in xenograft models suggests that innate immunity alone contributes to the efficacy of CD47-targeted therapy, but could also indicate that immune-independent mechanisms are involved. These could include CD47′s regulation of cell proliferation, survival, apoptosis, angiogenesis, migration, and metastasis [113,114]. Thus, attributing the effects of CD47 blockade solely to the inhibition of the phagocytosis checkpoint is an oversimplification, as its efficacy is likely determined by the net effect of pro- and anti-tumor mechanisms regulated by CD47. For this reason, an improved understanding and consideration of the signaling axes disrupted by CD47 blockade in tumor, immune, and other stromal cells is required to fully interpret its anti-tumor efficacy. Studies to define immune-independent mechanisms governed by CD47 that contribute to lung tumor sensitivity and resistance to CD47-targeted therapy are also warranted since they could inform rational therapeutic combinations as described in Section 7.4. 

### 7.2. Response-Predictive Biomarkers: How to Select Patients for CD47-Targeted Therapy? 

Biomarkers are important for choosing therapies most likely to be effective in a given patient population. Like PD-L1 expression for ICIs, CD47 positivity (CD47+) will likely be used to assign CD47 blockade. Implementing a clinical test for CD47 expression will require the development of a robust IHC assay, establishment of CD47+ thresholds associated with response (or lack thereof), validation of the assay’s utility in a large cohort of patients, and FDA approval to use it as a therapeutic indication. This process has proven challenging with respect to PD-L1 due to difficulties in reaching a consensus on the antibody used for IHC, tumor heterogeneity in PD-L1 expression, and imperfect correlations between expression and response [270]. Additional clinical factors and/or tumor markers may also be important for selecting patients. For example, other checkpoint molecules that suppress adaptive immunity may confer resistance to CD47 blockade, warranting their consideration. Deciphering malignancy-specific mechanisms influencing the efficacy of CD47 blockade may also nominate proteins that regulate tumor sensitivity and resistance as response-predictive biomarkers. To date, early phase trials designed to evaluate the safety and tolerability of CD47-targeted IO have not assessed biomarkers for predicting response. Further research is needed to identify putative biomarkers to assess as CD47-targeted therapies progress through later phases of clinical trials.

### 7.3. Mitigating Toxicity: How to Circumvent Challenges Associated with the CD47 Antigen Sink?

Both CD47 mAbs and SIRPα–Fc fusion proteins have generally been well tolerated in patients, but grade 3–4 toxicities due to CD47 blockade on RBCs, platelets, and neutrophils were not uncommon [267]. High-grade thrombocytopenia, neutropenia, and anemia occurred in 6.1%, 5.5%, and 4.6% of patients, respectively [267]. Although manageable, these adverse effects may limit efficacy by restricting the maximal tolerated dose. The CD47 antigen sink could also deplete antibodies in circulation and reduce the therapeutic load delivered to tumors. This could be a greater issue for solid cancers due to the inherent complexities of tumor stroma and vasculature that can impede antibody delivery, which might explain the lower efficacy of CD47 mAb in solid tumor patients [271]. Although some trials have confirmed CD47 saturation on cells in circulation following antibody administration [253,272,273], confirmation of antibody binding to cancer cells sampled from on-treatment biopsies would be required to confirm this hypothesis. At present, only one study has reported detection of therapeutic antibodies in tumor tissue from a single ovarian cancer patient [253].

For CD47 blockade to benefit cancer patients, strategies to widen its therapeutic window are required. To this end, several approaches have been developed. The CD47 mAb Magrolimab is initially administered using a priming dose to deplete aged RBCs susceptible to phagocytic clearance induced by CD47 blockade to avoid anemia during maintenance dosing [253,274]. Engineering tactics have been used to minimize the toxicities of SIRPα–Fc fusion proteins, as described above [259,260]. Bispecific antibodies are also likely to enhance the tumor selectivity of CD47 blockade. Methods for localized drug administration are also being developed that could be applied to mitigate toxicities associated with the systemic delivery of CD47-targeted IO [275]. Most simply, CD47 blockade could be administered intratumorally, an approach that impaired the growth of subcutaneous tumor models [228,237,244]. Clinically, this method is restricted to easily accessible tumors, but its efficacy was demonstrated by intralesional delivery of TTI-621 to treat cutaneous forms of T cell lymphoma [276]. Additionally, nanoparticle-based approaches for enhancing tumor-focused therapeutic delivery are also in development [277]. With respect to CD47 blockade, studies testing nanoparticles that co-deliver CD47-targeted siRNA or CD47 antibodies along with chemotherapy, targeted therapy, or immune agonists have shown promising results in preclinical models [278,279,280,281,282,283,284,285].

### 7.4. Therapeutic Combinations: What Standard of Care Therapies and Newly Discovered Targets Could Be Integrated with CD47 Blockade to Maximize Efficacy?

Preclinical and clinical trial data suggest that CD47 blockade is unlikely to be successful as a monotherapy because its efficacy requires the expression of pro-phagocytic signals on tumor cells [267,286]. Accordingly, many trials are evaluating CD47 blockade in combination with chemotherapy, targeted therapy, or other IOs known to induce “eat me” signals, increase tumor immunogenicity, and/or stimulate tumor immunity. Opsonizing cancer cells with antibodies targeting specific proteins upregulated in tumors tags them for clearance by the complement system, phagocytes, and/or NK cells [287], rationalizing combinations of therapeutic antibodies with CD47 blockade. Consistent with preclinical findings, this strategy has already proven effective in the clinic, evident by increased patient response rates when CD47 blockade is combined with the anti-CD20 antibody, rituximab, or the anti-HER2 antibody, trastuzumab, in patients with lymphoma or advanced solid cancers, respectively [254,256,274]. Combining CD47 and PD-1/CTLA-4 blockade is another strategy that could enhance anti-tumor activity by relieving additional brakes on tumor immunity. This is a rational approach for NSCLC, given that PD-1 blockade is a standard of care therapy and PD-L1 may be co-expressed with CD47 in some lung tumors [131]. Investigators have reported objective responses in patients treated with ALX148 and pembrolizumab, and with TTI-621 and nivolumab, raising excitement for the results of ongoing trials evaluating similar combinations [254,256].

Cytotoxic agents like chemotherapies and small molecule inhibitors (i.e., targeted therapy) as well as radiotherapy, which are all routinely used to treat NSCLC, can induce cell surface expression of the “eat me” signals, calreticulin and HMGB1, through a mechanism termed immunogenic cell death [288]. By inducing DNA damage, they can also stimulate type I interferon signaling in dendritic cells that engages adaptive tumor immunity [237]. Doxorubicin, cisplatin, and the EGFR tyrosine kinase inhibitor, Osimertinib, have been found to potentiate the efficacy of CD47 blockade by upregulating pro-phagocytic signals in preclinical tumor models [228,289,290,291]. These combinations are further rationalized by findings that CD47 can be induced by cytotoxic drugs and EGFR [110,130,222,228,292]. Ongoing clinical trials will determine whether the enhanced efficacy observed in tumor models translates to patients, but early results are encouraging [293]. Finally, future studies to further characterize the mechanism of action of CD47 blockade may reveal specific vulnerabilities exposed by CD47 loss-of-function, revealing additional targets for combination therapies. 

## 8. Perspective and Conclusions

Since most lung tumors are ineligible for or fail to respond to currently approved ICIs, lung cancer patients would benefit greatly from the availability of additional IO treatment options. A large body of preclinical evidence suggests that targeting CD47 has excellent therapeutic potential in NSCLC, particularly in *EGFR*-mutant tumors. This combined with the fact that CD47 is highly expressed in ≥30% of NSCLC emphasizes the clinical implications of CD47 blockade, suggesting it could be broadly applicable in lung cancer patients. Yet, clinical trials evaluating CD47 blockade in patients with solid malignancies including NSCLC have shown modest results. We are optimistic that further preclinical and clinical research will inform the most effective strategies for using CD47-targeted therapy to benefit NSCLC patients. To successfully translate CD47 blockade into lung cancer clinics, we propose that future research efforts should be focused on four key areas as outlined in Section 7 above: (1) understanding the mechanisms governing lung tumor sensitivity and resistance to CD47 inhibition; (2) discovering response-predictive biomarkers and establishing a robust clinical test for assessing CD47 expression in tumor tissues; (3) developing alternative approaches for administering CD47 blockade to avoid toxicity issues; and (4) identifying rational therapeutic combinations including those incorporating CD47-targeted therapy with standard of care lung cancer therapies. Addressing these knowledge gaps and making advancements in these critical areas will guide the development of CD47-targeted therapy and help propel it to become a next-generation IO for improving lung cancer outcomes.

## Figures and Tables

**Table 1:** CD47 expression and clinical relevance across various malignancies based on immunohistochemistry studies.

Lung Cancer				
Cancer Type	Sample Size	% CD47 Expression	Prognostic Association or Clinical Significance	References
Non-Small Cell Lung Cancer	169	84 (>1% cells) 65.7 (>50% cells)	Worse PFS and OS	[50]
	191	53.4 (>10% cells) 33 (>10% strong staining)	Worse OS and DFS	[125]
	80	57.5 (IRS ≥ 6)	Advanced TNM stage, metastasis, ≠	[126]
	384	49.7 (IRS ≥ 3)	Advanced TNM stage and histological subtype, tumor size; worse RFS	[127]
	84	50 (>median staining)	Worse OS	[128]
	98	29.6 (>50% cells)	Advanced tumor stage, €	[129]
	120	75 (IRS > 1)	LN metastasis, ≠	[130]
	430	68.8 (TPS > 5%)	Worse OS, TNM stage	[131]
Small-Cell Lung Cancer	104	84.6 (≥10% cells)	Worse OS and DFS	[132]
	29	27.5 (>1% cells)	None reported, €	[133]
**Other Cancers**				
**Cancer Type**	**Sample Size**	**% CD47 Expression**	**Prognostic Association or** **Clinical Significance**	**References**
Acute Myeloid Leukemia	171	94.7 (detectable staining) 18.7 (≥3)	Higher tumor load, €	[134]
Bladder Cancer	87	43.7 (IRS ≥ 1) 9.2 (IRS ≥ 8)	None reported, €	[135]
Breast Invasive Ductal Carcinoma	137	61.3 (≥0.8) ↟	Advanced TNM stage and histological grade; worse OS and DFS	[136]
Classical Hodgkin Lymphoma	16	100 (≥1+) 50 (≥2+)	None reported, ≠	[137]
Colorectal Cancer	468	53.4 (IRS >5)	Distant metastasis; advanced TNM stage; worse OS	[138]
	293	53 (IRS ≥4)	Advanced TNM stage; worse OS and DFS	[139]
Diffuse Large B-cell Lymphoma	55	54.5 (cutoff not specified)	Worse OS	[140]
	120	59 (>median staining)	MYC+, €	[141]
	238	63.1 (≥1) ↡ 16.4 (≥3) ↡	None reported, €	[142]
Endometrial Carcinoma	165	94.6 (>1% cells) 21.2 (>50% cells)	Advanced histological grade, €	[143]
Esophageal Squamous Cell Cancer	102	54.9 (>median cells)	LN metastasis; worse OS and disease-specific survival	[144]
Glioblastoma	46	82.6 (cutoff not specified)	Disease recurrence; worse OS	[145]
Hepatocellular Carcinoma	166	21.7 (>10% cells)	Advanced stage; frequent vessel invasion, €	[146]
	390	49.5 (>median cells)	Advanced TNM stage; increased vascular invasion; worse OS and RFS	[147]
Nasopharyngeal Carcinoma	66	56.1 (≥10% cells)	Worse OS; increased disease recurrence	[148]
Osteosarcoma	20	85 (cutoff not specified)	Metastasis; worse OS and PFS	[149]
Ovarian cancer	26	46.2 (≥2+)	Worse PFS	[150]
	116	90.5 (IRS ≥ 1) 60.3 (IRS ≥ 5)	Advanced stage; LN metastasis; worse OS	[151]
	86	91.9 (IRS ≥ 1) 60.5 (IRS ≥ 5)	Tumor stage; chemo-resistance; worse OS	[152]
Pancreatic Neuroendocrine Tumor	47	100 (H-score ≥ 1)	Disease recurrence/death; lymph node metastasis; lymph node and perineural invasion, *	[153]
Triple Negative Breast Cancer	57	77.2 (IRS ≥ 7)	Advanced TNM stage; LN invasion; increased recurrence; worse DFS	[154]

≠ No survival analysis was performed; € survival analysis conducted revealed no significant association with CD47. Details in brackets indicate criteria used to define CD47 expression in each study. ↟ Scored out of 3 based on staining intensity and percentage of positive tumor cells; ↡ Scored on 5 levels according to intensity (0, completely negative; 1, faint; 2, weak; 3, intermediate; 4, strong). * Clinical associations listed were found to be correlated with high CD47 expression in all studies except for pancreatic neuroendocrine tumors, in which low CD47 expression was associated with disease progression; IRS, immune reactive score (staining intensity x percentage of positive cells; 0–12); PFS, progression-free survival; OS, overall survival; DFS, disease-free survival; TNM, tumor node metastasis; RFS, recurrence-free survival; and LN, lymph node.

**Table 2:** CD47-targeted therapies being evaluated in ongoing and completed clinical trials including NSCLC patients.

Trial	Treatment(s)	Indication	Phase	Status
CD47 Monoclonal Antibody				
NCT04349969	AK117	Malignant Neoplasms	I	Completed
NCT05235542	AK117 ± AK104, Oxaliplatin, Cisplatin, Paclitaxel, Irinotecan, Docetaxel, 5-FU	Advanced Malignant Tumors	I, II	Ongoing
NCT05214482	AK117 + AK112 ± Chemotherapy	Advanced Malignant Tumors	I, II	Ongoing
NCT05229497	AK117 + AK112 ± Carboplatin, Cisplatin, 5-FU	Advanced Malignant Tumors	I, II	Ongoing
NCT03763149	IBI188	Advanced Malignancies	I	Ongoing
NCT05221385	Gentulizumab	Solid Tumors, Non-Hodgkin Lymphoma	I	Ongoing
NCT04900519	STI-6643	Solid Tumors, Relapsed Solid Neoplasms, Refractory Tumors	I	Ongoing
NCT05429008	HMPL-A83	Advanced Tumors	I	Ongoing
NCT05192512	TQB2928	Advanced Cancers	I	Ongoing
NCT03834948	AO-176 ± Paclitaxel, Pembrolizumab	Solid Tumors	I, II	Completed
NCT05276310	IMC-002	Advanced Cancer	I	Ongoing
NCT03512340	SRF231	Advanced Solid Cancers, Hematologic Cancers	I	Completed
NCT02216409	Hu5F9-G4 (Magrolimab)	Solid Tumors	I	Completed
**CD47–PD-L1 Bispecific Antibody**				
NCT05780307	IMM2520	Advanced Solid Tumors, NSCLC, SCLC, Breast Cancer, SCC Head and Neck, CRC	I	Ongoing
NCT05200013	BAT7104	Advanced Solid Tumors	I	Ongoing
NCT04881045	PF-07257876	NSCLC, SSC Head and Neck, Ovarian	I	Ongoing
NCT04886271	HX009	Advanced Solid Tumors	II	Ongoing
NCT04097769	HX009	Advanced Solid Tumors	I	Completed
NCT04980690	IBC0966	Advanced Malignant Tumors	I, II	Ongoing
**CD47–Mesothelin Bispecific Antibody**				
NCT05403554	NI-1801	Non-Squamous NSCLC, Epithelial Ovarian, TNBC	I	Ongoing
**SIRPα–HER2 Bispecific Antibody**				
NCT05076591	IMM2902	Advanced Solid Tumors, Advanced Breast Cancer, Advanced Gastric Cancer	I	Ongoing
**SIRPα–Fc Fusion Protein**				
NCT03013218	ALX148 (Evorpacept) ± Pembrolizumab, Trastuzumab, Rituximab, Ramucirumab + Paclitaxel, 5-FU + Cisplatin	Advanced Solid Tumors, Lymphoma	I	Ongoing
**SIRPα Monoclonal Antibody**				
NCT05765851	DS-1103a + T-DXd	Advanced Solid Tumors, Breast Cancer	I	Ongoing
NCT05615974	LM-101 ± Toripalimab, Rituximab, Envafolimab	Malignant Tumors	I, II	Ongoing

The first drug listed in the Treatment(s) column is the CD47 blockade agent. TNBC, Triple Negative Breast Cancer; SCC, Squamous Cell Carcinoma; and CRC, Colorectal Cancer. FU, Fluorouracil.
